# Role of Medication Reconciliation in Managing Bradycardia: A Rare Cause of Medication-Induced Sinus Bradycardia

**DOI:** 10.7759/cureus.88550

**Published:** 2025-07-22

**Authors:** Buthainah Alhwarat, Khalid Sawalha, Ibrahim Alshaghdali, Abdelmoniem Moustafa, Hakan Paydak

**Affiliations:** 1 Internal Medicine, University of Arkansas for Medical Sciences, Little Rock, USA; 2 Cardiovascular Disease, University of Arkansas for Medical Sciences, Little Rock, USA; 3 Cardiometabolic Medicine, University of Missouri-Kansas City School of Medicine, Kansas City, USA; 4 Clinical Cardiac Electrophysiology, University of Arkansas for Medical Sciences, Little Rock, USA; 5 Electrocardiology and Clinical Cardiac Electrophysiology, University of Arkansas for Medical Sciences, Little Rock, USA

**Keywords:** antiepileptic medications, arrhythmia, conduction abnormality, lacosamide, medication, sinus bradycardia

## Abstract

Sinus bradycardia is defined as a heart rate of less than 60 beats per minute (bpm), and it can be attributed to many etiologies, some of which are reversible, including but not limited to acute coronary syndrome, active infections, electrolyte imbalances, hypothyroidism, and medications. We report the case of a 64-year-old man who was admitted to the intensive care unit for the management of status epilepticus, where he developed new-onset, life-threatening sinus bradycardia, requiring placement on support with two inotropic agents (epinephrine and dopamine) due to persistent hemodynamic instability. Cardiology was consulted as a result, and after extensive workup, including review of past medical conditions, laboratory testing, and telemetry review, including that for reversible etiologies, we identified one of the antiepileptic agents on his medication list as the culprit of his sinus bradycardia. This case serves to contribute to existing, very limited, clinical data on its adverse cardiovascular effects.

## Introduction

Sinus bradycardia is a common cardiac conduction abnormality that can be a result of a variety of etiologies, including physiological and pathological processes. Common pathologies include ischemia, aging, metabolic/endocrine derangements, and rheumatological disease processes [1]. Reversible causes of sinus bradycardia include electrolyte imbalances, drugs and toxins, hypothermia, hypothyroidism, and medications [1]. The management and evaluation of sinus bradycardia typically begin with ruling out potentially reversible causes through detailed history taking, along with a review of the patient's medical history and current medications [2]. Furthermore, workup of sinus bradycardia includes obtaining a 12-lead electrocardiogram (EKG), which would reveal the cardiac rhythm associated with the bradycardia the patient is experiencing, often facilitating the determination of the underlying etiology and pathology [2]. An EKG can also reveal certain etiologies that can precipitate sinus bradycardia, including coronary artery disease [2]. However, there are occasions where EKGs may not allow the precise localization of the etiology of bradycardia; therefore, they should always be correlated to the clinical context in these cases. Laboratory testing is also a cornerstone of sinus bradycardia evaluation. This can include testing thyroid hormone levels, electrolytes, and drug levels like digoxin and beta-blocking agents [2].

We report a case of severe sinus bradycardia in a patient admitted to the intensive care unit (ICU), which persisted despite standard management and resolved only after the dose reduction of a centrally acting agent not typically associated with bradycardia. This highlights the importance of considering all pharmacologic agents in the differential diagnosis of bradyarrhythmias.

## Case presentation

We present the case of a 64-year-old man who was admitted to the ICU upon initial presentation for the management of status epilepticus and received aggressive treatment with a multi-agent antiepileptic regimen, including high-dose lacosamide, valproic acid, and levetiracetam.

**Figure 1:**
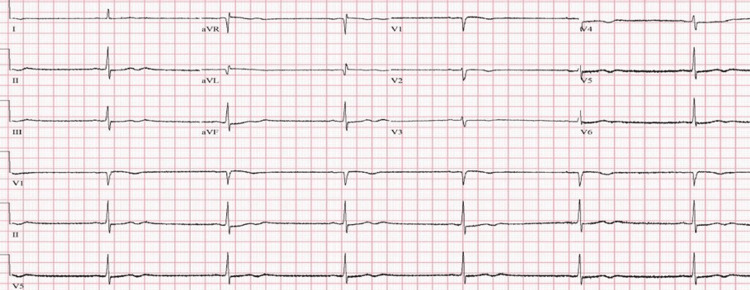
EKG obtained two days following the initial onset of bradycardia showing significant sinus bradycardia with a critical heart rate of 39 bpm EKG: electrocardiogram; bpm: beats per minute

**Figure 2:**
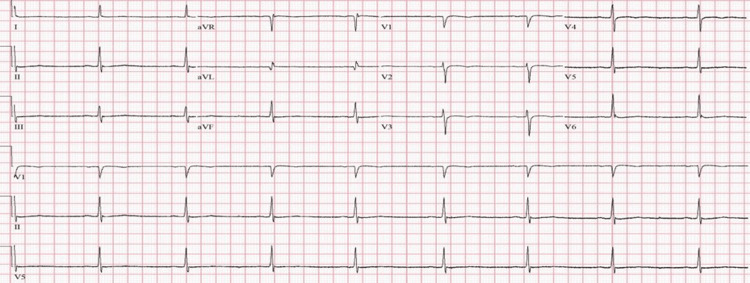
EKG obtained upon the initial onset of bradycardia showing sinus bradycardia rhythm with a heart rate of 48 bpm EKG: electrocardiogram; bpm: beats per minute

During his ICU stay, the patient developed new-onset, significant sinus bradycardia, with a heart rate noted at 30 beats per minute (bpm). He received three doses of 1 mg of atropine without improvement. Subsequently, he became hemodynamically unstable due to persistent hypotension from bradycardia, requiring inotropic support with dopamine and epinephrine infusions, which helped maintain adequate cardiac output at the time. Despite these interventions, his heart rate remained critically low, with a pulse of 30 bpm. Multiple EKGs confirmed sinus bradycardia (Figures 1-2). Transvenous pacing was considered the next step due to the persistent bradycardia despite inotropic therapy and repeated atropine doses. Given the complexity of the case, on day 2 of his ICU stay, the cardiology team was consulted for evaluation and guidance in the management of his persistent sinus bradycardia with hemodynamic instability.

A thorough cardiac workup was conducted, including a detailed review of the patient's past medical history, active problems, and current medication regimen. Reversible causes of sinus bradycardia, such as hypothyroidism and ischemia, were excluded. However, a review of his medication regimen raised concern that one of the drugs he was receiving could be the cause of his bradyarrhythmia. As a result, plans to initiate transvenous pacing were deferred due to the identification of a potential etiology.

**Figure 3:**
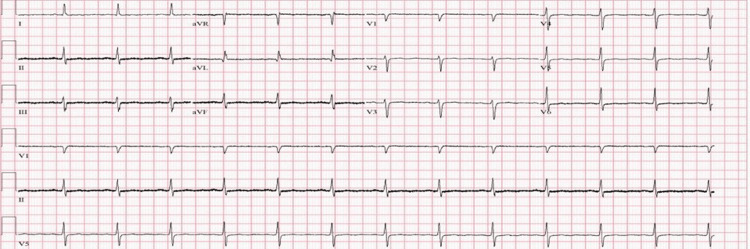
EKG obtained on initial admission to the ICU showing normal sinus rhythm with a heart rate of 78 bpm EKG: electrocardiogram; bpm: beats per minute; ICU: intensive care unit

After careful consideration and a review of the literature on side effect profiles, his sinus bradycardia was attributed to the antiepileptic drug lacosamide, which he was receiving for ongoing status epilepticus. Notably, lacosamide was one of his home medications for seizure disorder, along with levetiracetam and valproic acid, both of which were also continued during his ICU admission. A review of EKGs prior to ICU admission showed a normal heart rate and rhythm (Figure 3). Additionally, a chart review of encounters as recent as one month prior revealed no history of sinus bradycardia.

During his ICU stay, he received 1 g of IV levetiracetam daily and 3 g of valproic acid daily, equal to and 1.5 times higher than his home doses, respectively. Neurology was involved and informed about the identification of lacosamide as the likely causative agent of the significant sinus bradycardia, especially since it was administered at higher doses than his regular home dose. However, given his critical condition and ongoing seizure activity on electroencephalogram (EEG), neurology initially advised against changing the dose.

Due to the persistence of sinus bradycardia, which led to hemodynamic instability, and after multidisciplinary discussions, the neurology team agreed to adjust his antiepileptic regimen. This was the final attempt to address his persistent bradycardia before considering permanent pacemaker implantation. Upon initial ICU admission, he had been receiving 400 mg of IV lacosamide daily. Following the onset of bradycardia that was unresponsive to atropine, he was placed on inotropic support with dopamine and epinephrine. On day 2 of bradycardia onset, and after weighing risks and benefits with the cardiology and neurology teams, the lacosamide dose was reduced to 200 mg IV daily, half the original dose.

**Figure 4:**
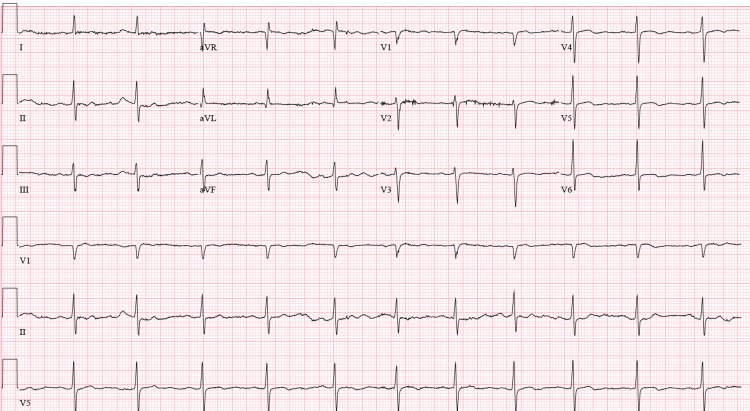
EKG obtained following lacosamide dose adjustment showing normal sinus rhythm with a heart rate of 66 bpm EKG: electrocardiogram; bpm: beats per minute

By day 5, inotropic agents were discontinued. His heart rate improved from the 30s to the 70s bpm over four days, indicating complete resolution of his conduction abnormality. He did not require cardiac pacing or repeated atropine administration (Figures 3-4). On day 6 after the initial bradycardia onset, following stabilization, he was transferred out of the ICU within two days to continue seizure management on the medical floor. He was scheduled for outpatient cardiology follow-up after discharge. At follow-up, the patient maintained normal sinus rhythm, and lacosamide was discontinued indefinitely.

## Discussion

The etiology of sinus bradycardia in acutely ill hospitalized patients, especially those admitted to the ICU with multiple simultaneous medical problems and receiving complex treatment regimens, can often be difficult to pinpoint. Many medication classes are known to cause cardiac arrhythmias, and antiepileptic agents, which are often overlooked in this context, are no exception. Our case report highlights lacosamide as the likely culprit in profound sinus bradycardia, particularly when used in high doses, such as those administered for IV loading, which can cause hemodynamic instability. Furthermore, it emphasizes the importance of conducting a thorough workup of reversible causes of sinus bradycardia and considering less commonly recognized etiologies to avoid unnecessary pacemaker implantation.

As a third-generation antiepileptic drug first approved by the US Food and Drug Administration (FDA) in 2008 for the management of focal seizures [3], lacosamide's mechanism of action is believed to involve selective enhancement of the slow inactivation of voltage-gated sodium channels, thereby reducing sodium influx into neuronal cells and decreasing their excitability [4]. Through this mechanism, lacosamide may delay conduction in myocardial tissue, particularly within the atrial conduction system.

While the efficacy of lacosamide as an antiepileptic agent has been demonstrated in various studies, like other anticonvulsants, it has been associated with serious adverse events, including cardiac complications [5]. Cardiac side effects reported in the literature include rhythm abnormalities such as sinus bradycardia, atrioventricular block, atrial fibrillation, atrial flutter, and ventricular tachycardia [6,7]. However, existing literature, including both experimental studies and clinical trials, has only limited documentation of clinically significant arrhythmias caused by lacosamide.

Lacosamide is known to have a long half-life, approximately 13 hours in younger individuals and 14-16 hours in the elderly. The incidence of cardiac adverse events is believed to increase in a dose-dependent manner [8,9]. Although sinus bradycardia is a less commonly reported cardiac adverse event, it has been documented in fewer than 1% of lacosamide users. According to McLaughlin et al., sinus bradycardia has been observed in 0.7-2.6% of patients treated with IV lacosamide [10]. In contrast, a review by Yadav et al. identified ventricular tachycardia as the most frequent and potentially life-threatening cardiac arrhythmia linked to this medication [11].

An important observation noted by multiple researchers is that IV loading doses of lacosamide are often the primary trigger for rhythm abnormalities [7]. A Korean systematic review reported the following arrhythmias associated with lacosamide: 17.6% complete heart block, 11.8% second-degree atrioventricular block, and 11.8% sinus pauses [9]. Furthermore, advanced age and a history of cardiac disease have been identified as additional risk factors for developing cardiac side effects during IV lacosamide therapy [9].

In contrast, oral lacosamide at recommended maintenance doses (≤400 mg/day) may still cause dose‑dependent PR prolongation; however, bradyarrhythmias are far less common and typically observed in patients with other risk factors, such as concomitant sodium channel‑blocking drugs or pre-existing conduction disease [12].

The most current evidence regarding the cardiac adverse effects of lacosamide comes from case reports. Only a few reports in the literature describe sinus node dysfunction in patients receiving lacosamide. Thus, our case report contributes to this limited body of work, as our patient experienced sinus bradycardia likely secondary to a high IV loading dose. However, more research is needed to clarify the causal relationship between lacosamide and its potential proarrhythmic effects, as current evidence is largely anecdotal. While the absence of a drug re-challenge and the presence of polypharmacy limit definitive attribution, we aim to contribute to the clinical understanding of lacosamide's potential proarrhythmic profile. Additionally, we highlight the importance of a meticulous inpatient approach in identifying reversible causes of bradycardia. In this case, a comprehensive review of the patient's history and medication list led to the identification of the causative agent and successful reversal of sinus bradycardia.

## Conclusions

Sinus bradycardia is a common cardiac rhythm abnormality that can arise from various etiologies, many of which are reversible. Our case report underscores the importance of conducting a thorough workup to rule out reversible causes before pursuing invasive treatments. We present the case of a 64-year-old man who developed medication-induced sinus bradycardia secondary to IV lacosamide therapy. The resolution of the patient's bradycardia following dose reduction, thereby avoiding unnecessary interventions such as pacemaker placement, reinforces the need for vigilance in identifying reversible causes through careful review of medical history and medication lists, with particular attention to home medications and any dose escalations. Finally, it may be beneficial to monitor patients initiated on lacosamide therapy, especially at higher IV doses, with continuous cardiac telemetry to detect potential arrhythmias.
